# Applications of deep convolutional neural networks to digitized natural history collections

**DOI:** 10.3897/BDJ.5.e21139

**Published:** 2017-11-02

**Authors:** Eric Schuettpelz, Paul B. Frandsen, Rebecca B. Dikow, Abel Brown, Sylvia Orli, Melinda Peters, Adam Metallo, Vicki A. Funk, Laurence J. Dorr

**Affiliations:** 1 National Museum of Natural History, Smithsonian Institution, Washington, DC, United States of America; 2 Office of the Chief Information Officer, Smithsonian Institution, Washington, DC, United States of America; 3 NVIDIA, Santa Clara, CA, United States of America

**Keywords:** convolutional neural networks, deep learning, machine learning, mass digitization, natural history collections

## Abstract

Natural history collections contain data that are critical for many scientific endeavors. Recent efforts in mass digitization are generating large datasets from these collections that can provide unprecedented insight. Here, we present examples of how deep convolutional neural networks can be applied in analyses of imaged herbarium specimens. We first demonstrate that a convolutional neural network can detect mercury-stained specimens across a collection with 90% accuracy. We then show that such a network can correctly distinguish two morphologically similar plant families 96% of the time. Discarding the most challenging specimen images increases accuracy to 94% and 99%, respectively. These results highlight the importance of mass digitization and deep learning approaches and reveal how they can together deliver powerful new investigative tools.

## Introduction

Deep learning can greatly surpass conventional machine learning by incorporating multi-layered neural networks capable of processing natural data in their raw form (LeCun et al. 2015). Deep convolutional neural networks (CNNs) are especially well suited to image classification and may even achieve superhuman performance (He et al. 2015). Already, CNNs are playing important roles in healthcare, speech recognition, and driverless cars. Natural history collections (NHCs) also benefit society in numerous ways, most notably supporting public health, safety, and agriculture (Suarez and Tsutsui 2004). NHCs are likewise fundamental to understanding biodiversity and they underlie studies of evolution, habitat loss, biological invasion, and climate change. The billions of specimens in NHCs could undoubtedly provide even greater social and scientific insight, but their data are typically accessible only to researchers who can physically visit repositories. Digitization efforts obviate the need for many types of in-person data gathering (Beaman and Cellinese 2012) and remarkable progress is now being made in compiling specimen data and images (Barkworth and Murrell 2012). Coupling these data with the classification capabilities of CNNs will unlock more of the rich potential of NHCs.

Deep learning might ultimately be leveraged in many ways for many different types of NHCs. Here, we focus on the digitized portion (currently 1.2 million specimens) of the United States National Herbarium. Our analyses, focused on the detection of specimens previously treated with mercury and the discrimination of superficially similar plant families, are complementary to those recently published on species identification (Carranza-Rojas et al. 2017) and further demonstrate how CNNs might be used to learn more from NHCs.

## Materials and methods

To assess the potential of using CNNs to classify specimen images obtained from NHCs, we assembled two distinct datasets. Both datasets contained two image categories, with an approximately equal number of images in each category. Some specimen images were obtained with a traditional light box, but most were acquired via a conveyor system managed by the Smithsonian Digitization Program Office.

In the past, mercuric chloride was sometimes used by collectors or repositories to prevent insect damage to specimens. Unfortunately, this substance is also toxic to humans and knowing the number and location(s) of contaminated specimens in a collection is important. One can test for mercury vapor in herbarium cabinets (Hawks et al. 2004), but it is also possible to visualize contamination as chemical reactions of mercury with mounting paper and air leave a distinctive stain (images of unstained and stained specimens are provided in Fig. 1). Such staining, which can vary in severity and location, is rarely recorded in specimen metadata. Therefore, to assess the utility of using deep learning to identify mercury-contaminated specimens, we manually assembled a set of 7,777 unstained (https://doi.org/10.6084/m9.figshare.5423098) and 7,777 stained (https://doi.org/10.6084/m9.figshare.5423083) images.

The automated identification of specimens could make a valuable contribution to biological research (Gaston and O'Neill 2004). However, in botany, applications to date have generally been restricted to living plants and almost all studies have employed conventional machine learning approaches requiring considerable preprocessing (e.g., Unger et al. 2016). Deep learning approaches offer significant advantages and may bring greatly improved accuracy (Carranza-Rojas et al. 2017). To evaluate the capabilities of CNNs to discriminate among plant taxa in our herbarium, we assembled a data set focused on two closely related families: clubmosses (Lycopodiaceae) and spikemosses (Selaginellaceae). Although these two families differ in microscopic features (clubmosses are homosporous and have nonligulate leaves, whereas spikemosses are heterosporous and have ligulate leaves), they are superficially similar (Fig. 1). Our dataset included 9,276 clubmoss (https://doi.org/10.6084/m9.figshare.5423176) and 9,113 spikemoss (https://doi.org/10.6084/m9.figshare.5423182) images.

CNNs were built in Mathematica version 11.1 (Wolfram Research Inc.) and trained on NVIDIA K80 GPUs. For each dataset (stained/unstained and clubmoss/spikemoss), we randomly partitioned the images into three non-overlapping sets each time before training the network: 70% were used for training the model; 20% were used for validation; and 10% were reserved as our test dataset (i.e., the images used to train the CNNs were not used to assess their accuracy). We resized the color images to 256×256 pixels, creating a 3×256×256 tensor for our input layer (the first dimension separated by RGB values), and explored the performance of a variety of CNNs for each dataset. For the stained/unstained dataset, the best CNN included four convolutional and four pooling layers (Table 1; https://doi.org/10.6084/m9.figshare.5501743). For the clubmoss/spikemoss dataset, the best CNN included two convolutional layers and two pooling layers (Table 2; https://doi.org/10.6084/m9.figshare.5501716). The code used to define and train these CNNs can be found in our Mathematica notebooks (Suppl. materials 1, 3, 2, 4).

## Results and discussion

Our best performing CNNs were remarkably effective in distinguishing stained from unstained specimens, as well as clubmosses from spikemosses (Fig. 2). Images withheld from training were correctly identified 90% and 96% of the time, respectively. Misclassifications were roughly symmetrical. If the most difficult images to classify (the 10% with classification probabilities closest to 0.5) were removed from the test set, accuracy jumped to 94% and 99%, respectively.

The present study demonstrates two different ways in which CNNs can be applied to NHCs. The mercury staining analysis has practical implications for collections management, while the analysis centered on distinguishing families is interesting from both collections management and research perspectives. Our stained vs. unstained network could theoretically be applied to digitized specimens in other herbaria to help identify mercury hotspots for potential remediation. Likewise, our family discrimination network has the potential to be further developed into a universal tool to identify unknowns or to flag specimens in need of additional study, in the United States National Herbarium and in other NHCs.

Our work highlights the importance of proper metadata curation when approaching a machine learning project. Assembling the training dataset for the mercury analysis required many person hours to visually inspect images for staining, whereas clubmoss and spikemoss images were easily compiled using specimen metadata alone. Nascent efforts in digitization in NHCs must carefully consider the acquisition and curation of metadata because it affects how quickly machine learning tools can be applied to digitized museum collections.

## Supplementary Material

Supplementary material 1 Notebook used to define and train the unstained/stained CNN. Data type: Mathematica notebook
File: oo_162582.nb Paul B. Frandsen, Abel Brown

Supplementary material 2 Annotated notebook used to define and train the unstained/stained CNN. Data type: PDF
File: oo_162585.pdf Paul B. Frandsen, Abel Brown

Supplementary material 3 Notebook used to define and train the clubmoss/spikemoss CNN. Data type: Mathematica notebook
File: oo_162583.nb Paul B. Frandsen, Abel Brown

Supplementary material 4 Annotated notebook used to define and train the clubmoss/spikemoss CNN Data type: PDF
File: oo_162589.pdf Paul B. Frandsen, Abel Brown

## Figures and Tables

**Figure 1a.**
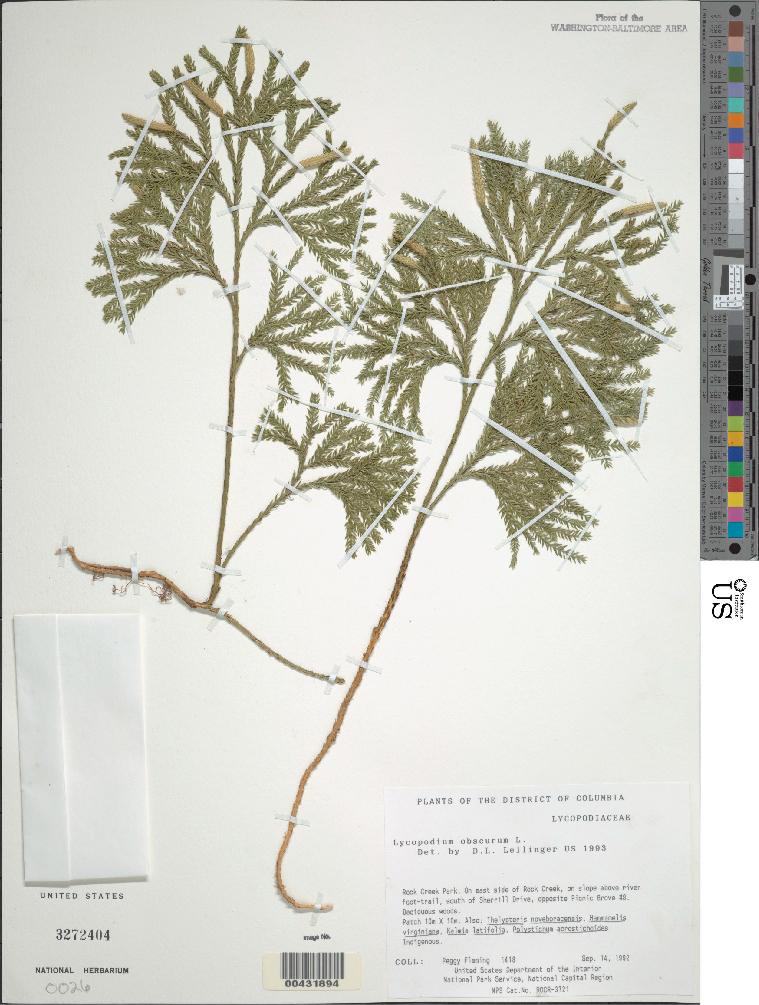
Unstained clubmoss.

**Figure 1b.**
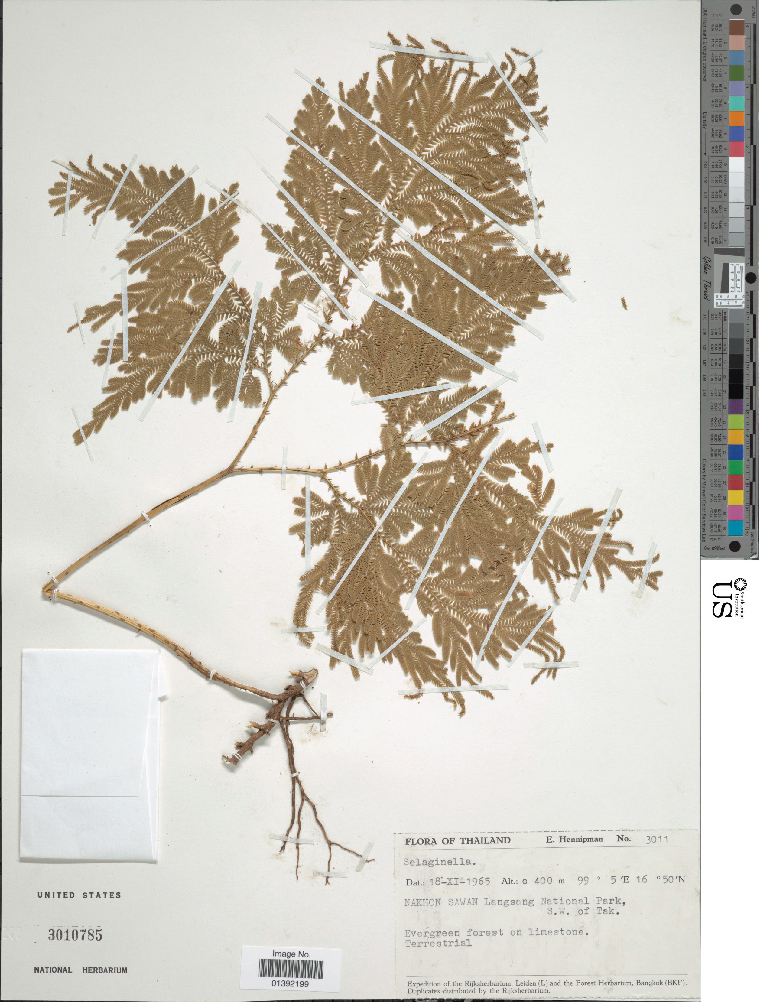
Unstained spikemoss.

**Figure 1c.**
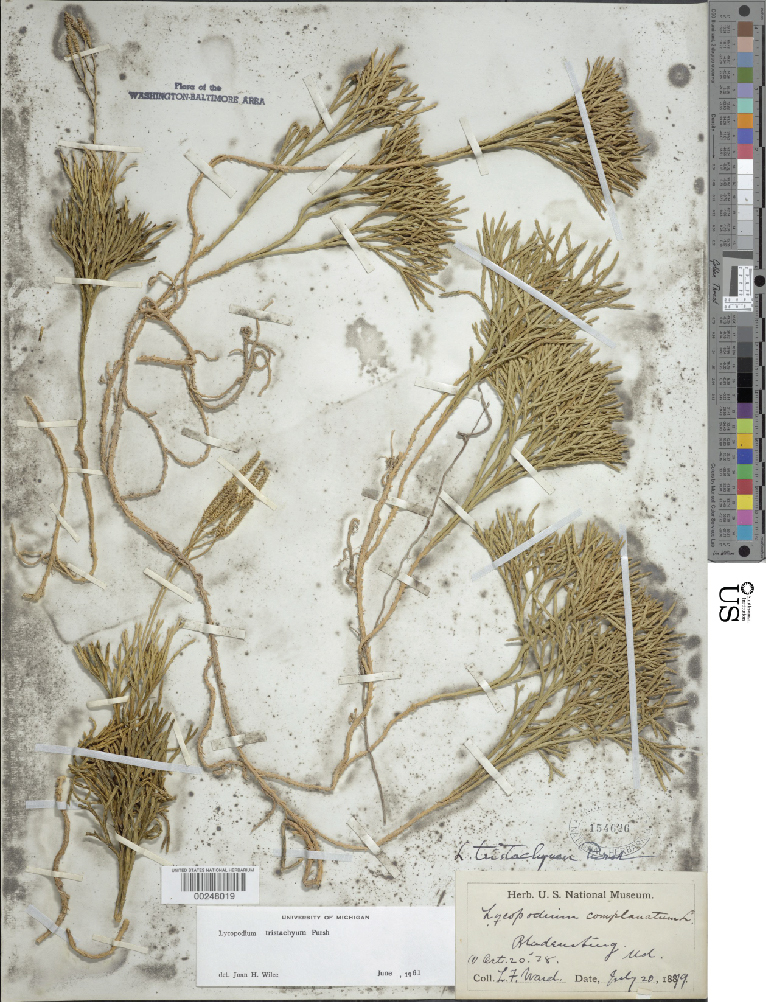
Stained clubmoss.

**Figure 1d.**
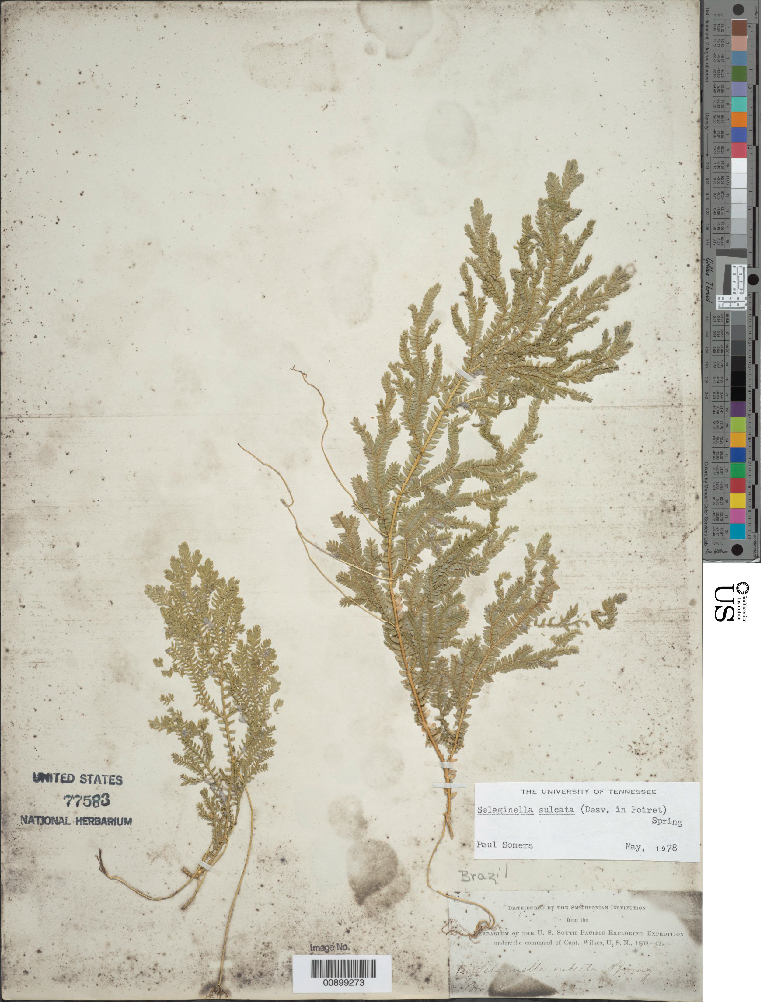
Stained spikemoss.

**Figure 2a.**
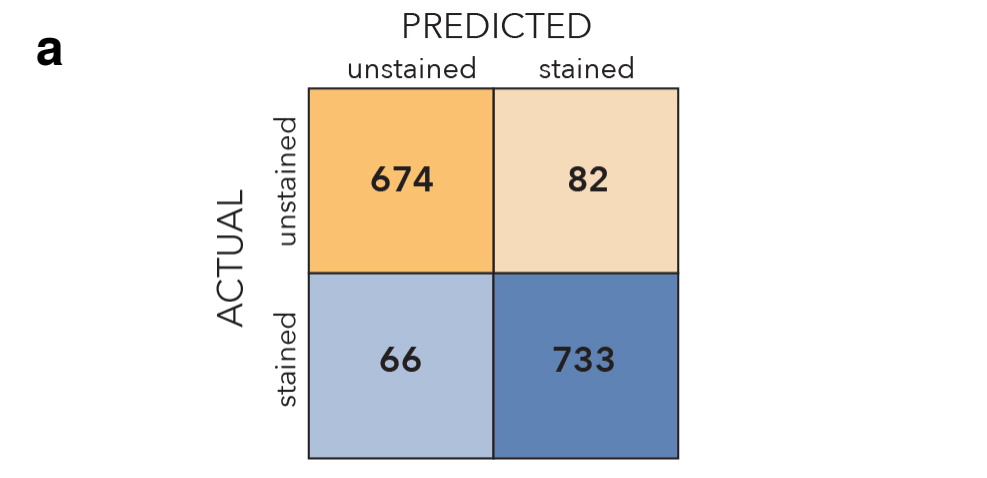
Unstained/stained confusion matrix.

**Figure 2b.**
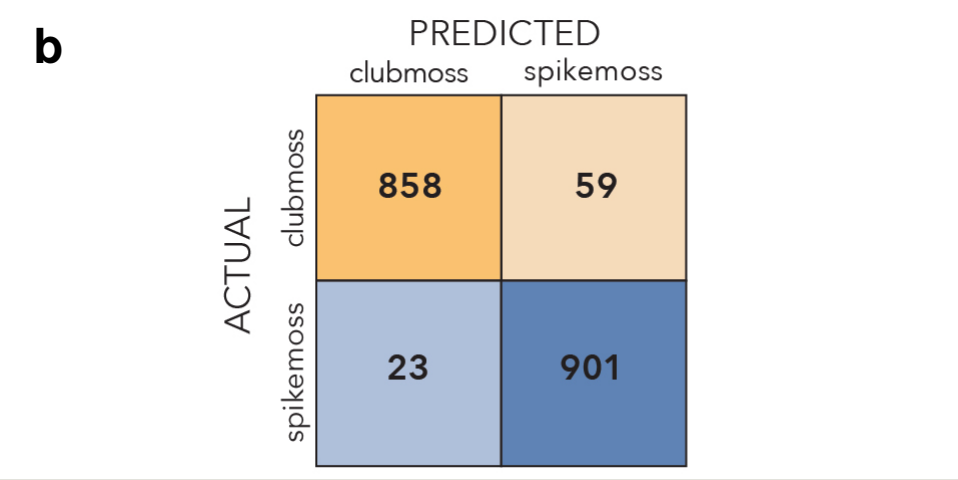
Clubmoss/spikemoss confusion matrix.

**Figure 2c.**
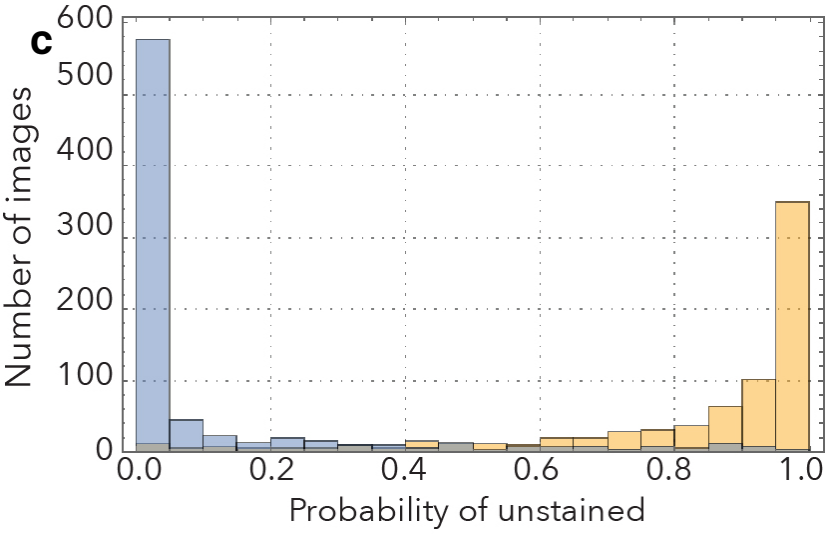
Unstained/stained probability distribution.

**Figure 2d.**
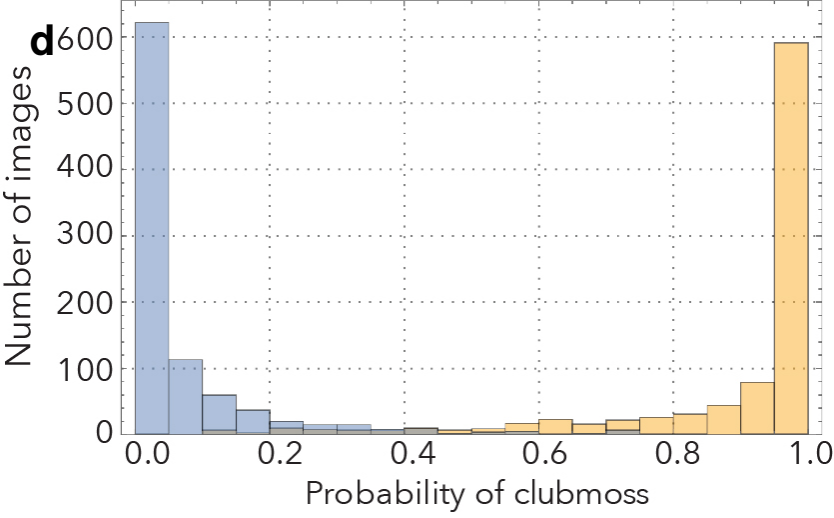
Clubmoss/spikemoss probability distribution.

**Figure 2e.**
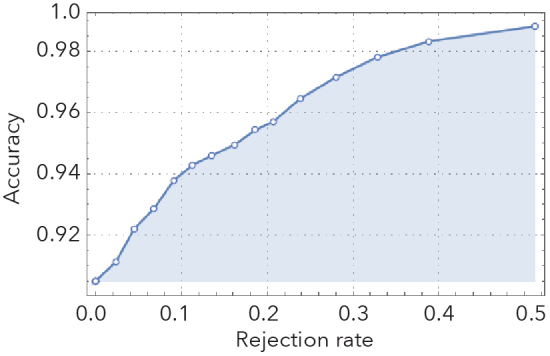
Unstained/stained rejection plot.

**Figure 2f.**
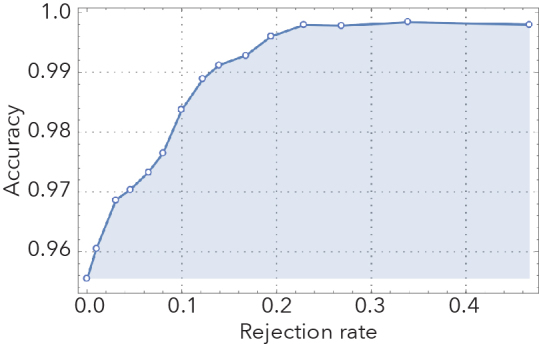
Clubmoss/spikemoss rejection plot.

**Table 1.** Constitutive layers and tensor/vector shapes for the unstained/stained CNN.

Layer	Type	Shape
Input	3-tensor	3×256×256
ConvolutionLayer	3-tensor	16×252×252
BatchNormalizationLayer	3-tensor	16×252×252
Ramp (ReLU)	3-tensor	16×252×252
PoolingLayer	3-tensor	16×126×126
ConvolutionLayer	3-tensor	32×122×122
BatchNormalizationLayer	3-tensor	32×122×122
Ramp (ReLU)	3-tensor	32×122×122
PoolingLayer	3-tensor	32×61×61
ConvolutionLayer	3-tensor	64×57×57
BatchNormalizationLayer	3-tensor	64×57×57
Ramp (ReLU)	3-tensor	64×57×57
PoolingLayer	3-tensor	64×28×28
ConvolutionLayer	3-tensor	48×26×26
BatchNormalizationLayer	3-tensor	48×26×26
Ramp (ReLU)	3-tensor	48×26×26
PoolingLayer	3-tensor	48×13×13
FlattenLayer	vector	8112
DropoutLayer	vector	8112
LinearLayer	vector	500
Ramp (ReLU)	vector	500
LinearLayer	vector	2
SoftmaxLayer	vector	2
Output	class	

**Table 2.** Constitutive layers and tensor/vector shapes for the clubmoss/spikemoss CNN.

**Layer**	**Type**	**Shape**
Input	3-tensor	3×256×256
ConvolutionLayer	3-tensor	10×252×252
BatchNormalizationLayer	3-tensor	10×252×252
Ramp (ReLU)	3-tensor	10×252×252
PoolingLayer	3-tensor	10×126×126
ConvolutionLayer	3-tensor	40×122×122
BatchNormalizationLayer	3-tensor	40×122×122
Ramp (ReLU)	3-tensor	40×122×122
PoolingLayer	3-tensor	40×61×61
FlattenLayer	vector	148840
DropoutLayer	vector	148840
LinearLayer	vector	500
Ramp (ReLU)	vector	500
LinearLayer	vector	2
SoftmaxLayer	vector	2
Output	class	
