# Supplementary material for: Applications of deep convolutional neural networks to digitized natural history collections
Source: Biodivers Data J. 2017 Nov 2;(5):e21139. doi: 10.3897/BDJ.5.e21139 (PMC5680669; doi:10.3897/BDJ.5.e21139)
Supplement: Supplementary material 2 — Annotated notebook used to define and train the unstained/stained CNN. [file bdj-05-e21139-s002.pdf]

Load the paths and the data

```
root = "/pool/isilon/genomics/frandsenp/NVIDIA/Botany/Mathematica";
datadir = FileNameJoin[{root, "data"}];
cleandir = "/pool/isilon/genomics/frandsenp/NVIDIA/smaller_clean";
contaminateddir = "/pool/isilon/genomics/frandsenp/NVIDIA/contam_test_256";
cleanfiles = FileNames["*.jpg", cleandir];
contaminatedfiles = FileNames["*.jpg", contaminateddir];
{Length@cleanfiles, Length@contaminatedfiles}
```

```
{7777, 7777}
```

Configure the notebook to use the GPU and properly use Java functions on Linux

```
Needs["CUDALink`"]
```

```
<< JLink`;
InstallJava[];
ReinstallJava[JVMArguments → "-Xmx10g"];
```

Import the data

```
cleandat = ParallelMap[Import[#] &, cleanfiles];
contamdat = ParallelMap[Import[#] &, contaminatedfiles];
```

Take a look at a few random pictures to make sure they imported properly

```
RandomChoice@contamdat
```

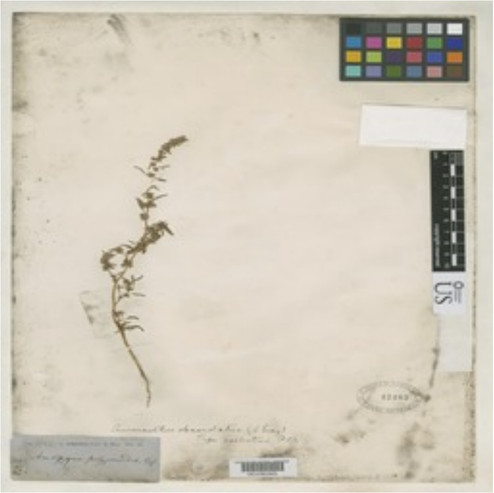

```
RandomChoice@cleandat
```

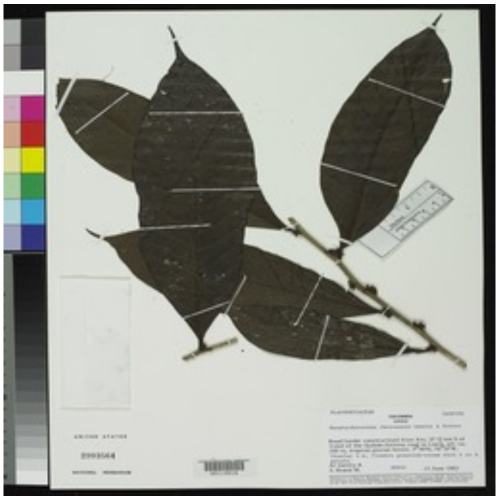

Make sure that the number of images properly loaded

```
{Dimensions@cleandat, Dimensions@contamdat}
```

```
RandomSample[cleandat, 5]
```

{ 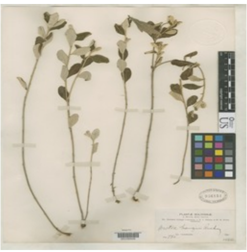 , 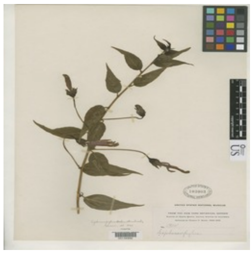 ,

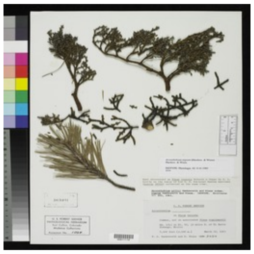 , 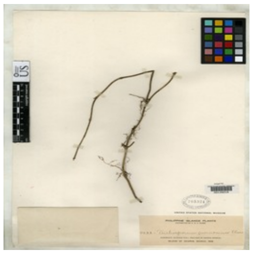 , 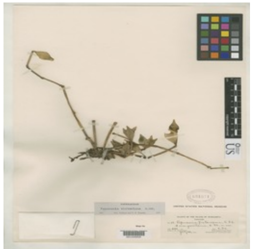 }

```
RandomSample[contamdat, 5]
```

{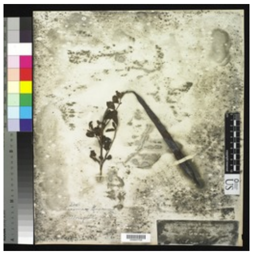, 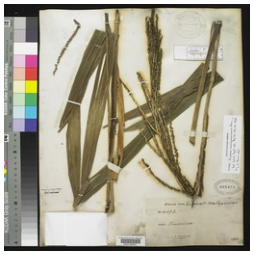,

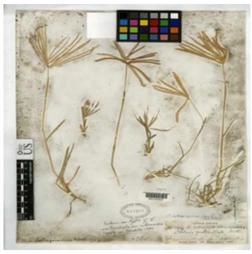, 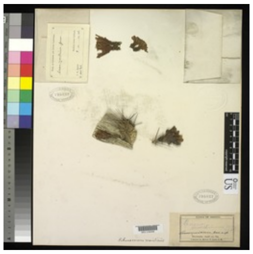, 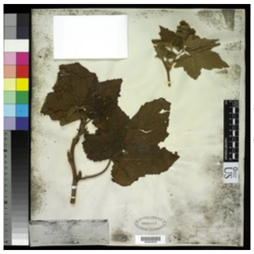}

Flatten out the data and label the images

```
dat = RandomSample@
   Join[Thread[cleandat → "clean"], Thread[contamdat → "contaminated"]];
Dimensions@
 dat
```

```
{15 554}
```

Take another look to ensure the annotations look correct

```
RandomSample[dat, 30]
```

{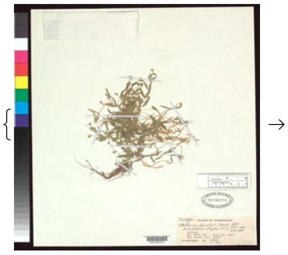 → clean, 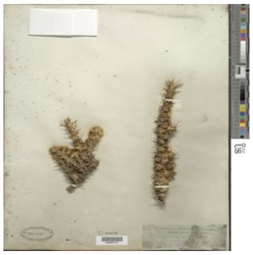 → contaminated,

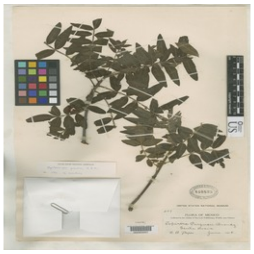 → clean, 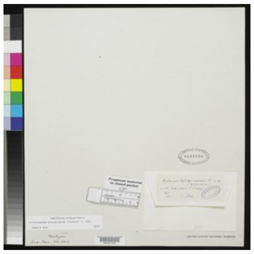 → clean,

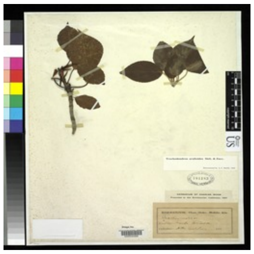 → contaminated, 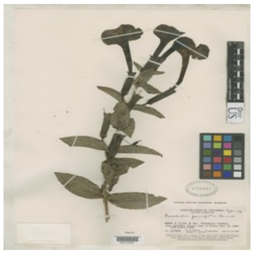 → clean,

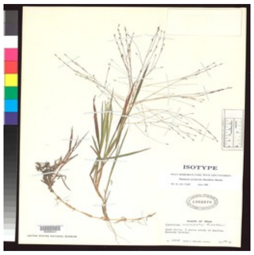

→ clean, 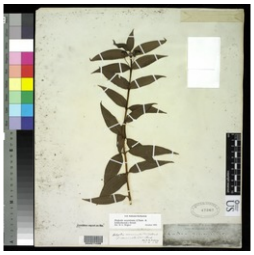 → contaminated,

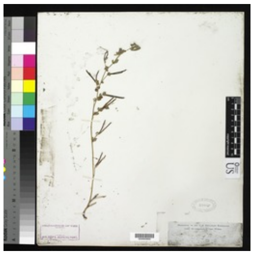 → contaminated, 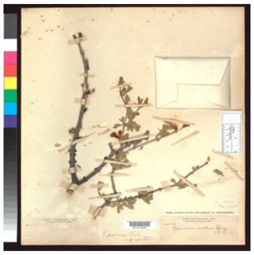 → contaminated,

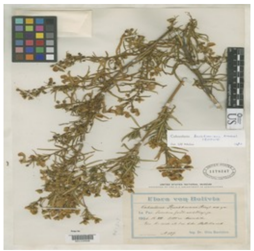 → clean, 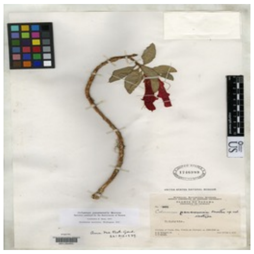 → clean,

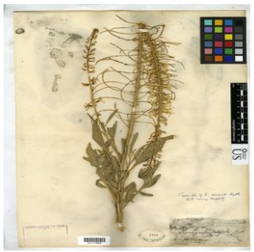 → contaminated, 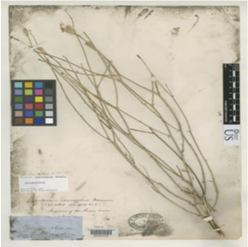 → contaminated,

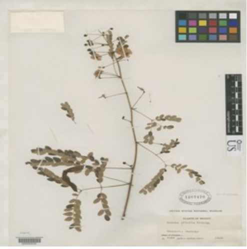 → clean, 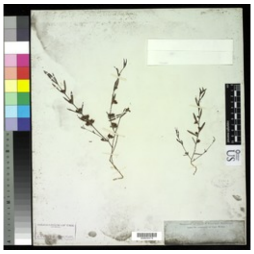 → contaminated,

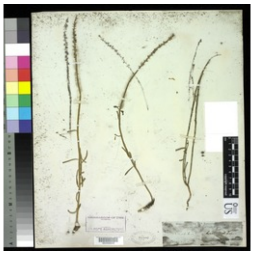 → contaminated, 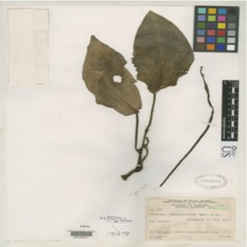 → clean,

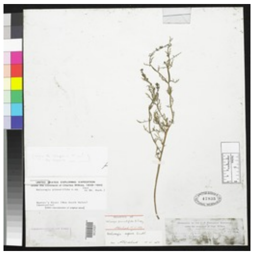 → contaminated, 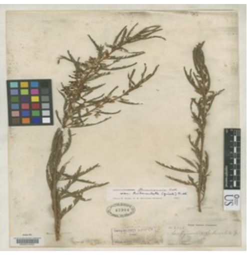 → contaminated,

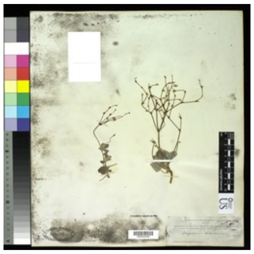 → contaminated, 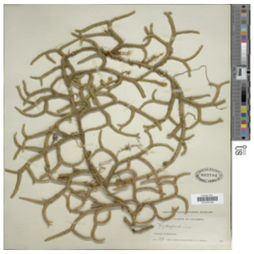 → clean,

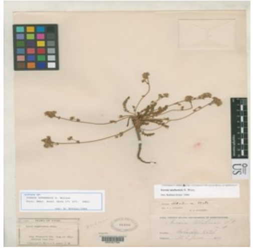 → clean, 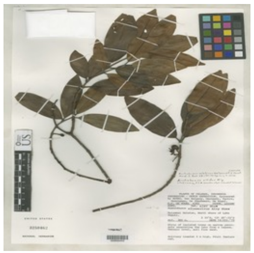 → clean, 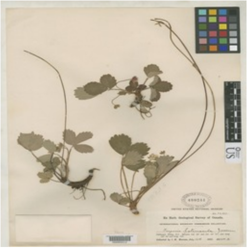 → clean,

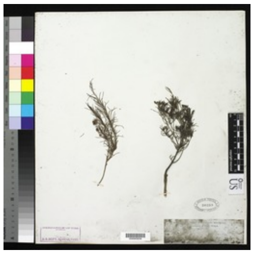 → contaminated, 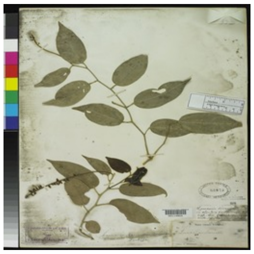 → contaminated,

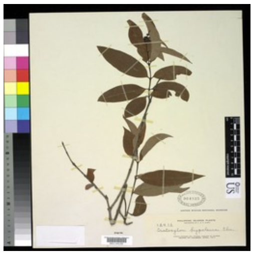 → clean, 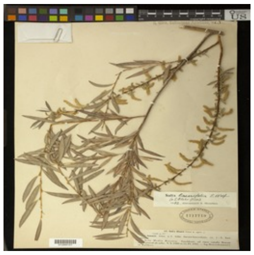 → clean, 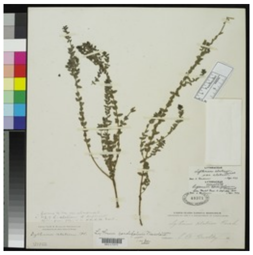 → clean}

Estimate image entropy, contaminated images should have, on average, higher entropies

```
clnEntropy = ParallelMap[ImageMeasurements[#, "Entropy"] &, cleandat];
conEntropy = ParallelMap[ImageMeasurements[#, "Entropy"] &, contamdat];
```

```
Histogram[{clnEntropy, conEntropy}, 40, ChartLegends → {"Clean", "Contaminated"},
 GridLines → Automatic, GridLinesStyle → Directive[Dotted, Gray],
 Frame → True, FrameLabel → {Style["Entropy", FontSize → 14]}]
```

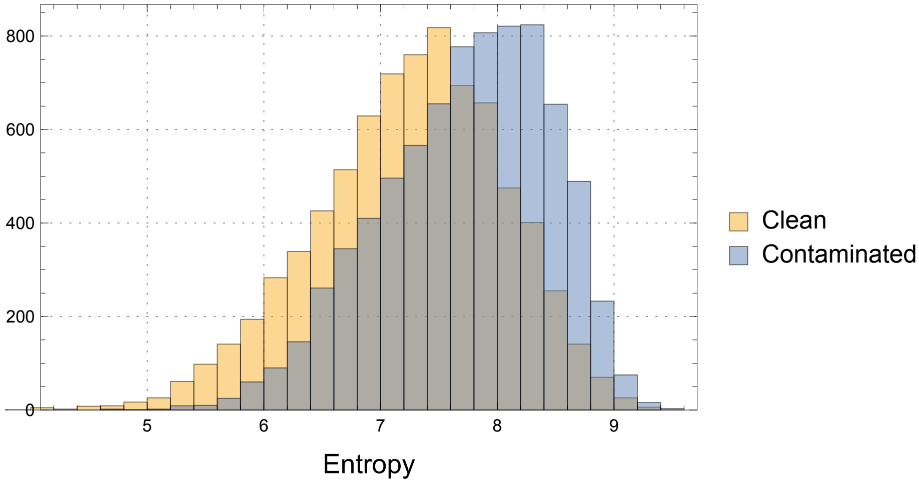

```
Histogram[
 {Map[Mean, Partition[clnEntropy, 108]], Map[Mean, Partition[conEntropy, 108]]},
 40, ChartLegends → {"Clean", "Contaminated"},
 GridLines → Automatic, GridLinesStyle → Directive[Dotted, Gray],
 Frame → True, FrameLabel → {Style["Entropy", FontSize → 14]}]
```

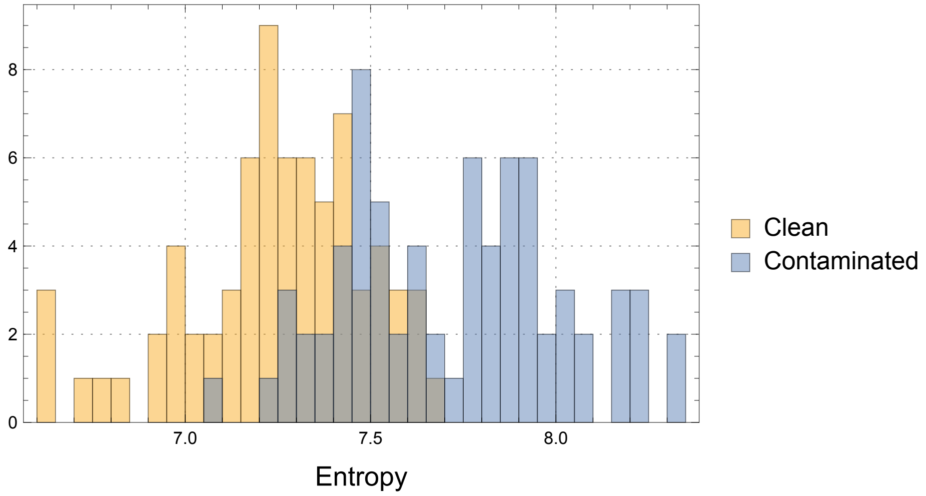


```
Plot[{CDF[EmpiricalDistribution[Map[Mean, Partition[conEntropy, 108]]], x],
  CDF[EmpiricalDistribution[Map[Mean, Partition[clnEntropy, 108]]], x]},
 {x, 6.4, 8.4}, PlotLegends → {"Contaminated", "Clean"},
 GridLines → Automatic, GridLinesStyle → Directive[Dotted, Gray],
 Frame → True, FrameLabel → {Style["Entropy", FontSize → 14]}]
```

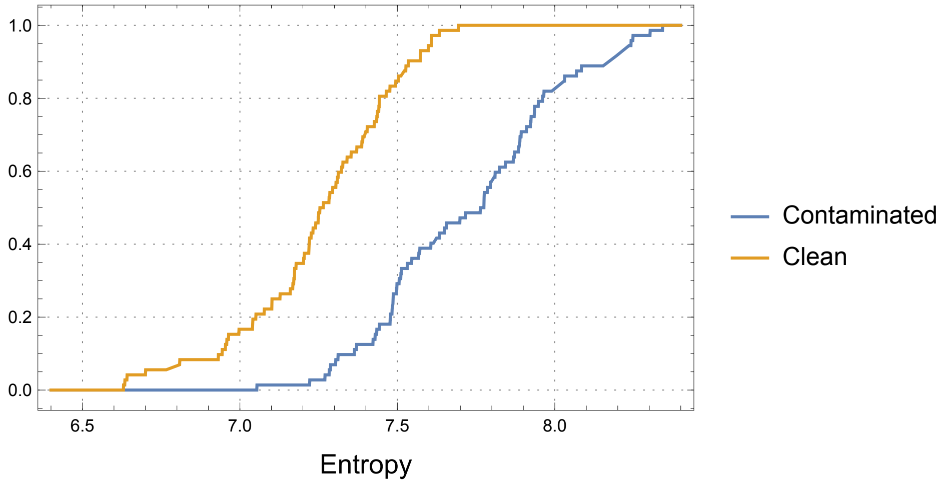

Randomly order images, then generate training data set (70% of data, “tix”), validation data set (20% of data, “vix”), and test data set (10% of data, “ttix”). Note that the test data set will not be used for training on the neural net.

```
rs = RandomSample[Range[Length[dat]]];
tix = rs[[1 ;; Round[Length[dat] * 0.7]]];
vix = rs[[Round[Length[dat] * 0.7] + 1 ;; Round[Length[dat] * 0.9]]];
ttix = rs[[Round[Length[dat] * 0.9] + 1 ;;]];
```

```
{Length@tix, Length@vix, Length@ttix}
```

```
{10 888, 3111, 1555}
```

Generate architecture for untrained neural net

```
net = NetChain[
  {
   ConvolutionLayer[16, {5, 5}],
   BatchNormalizationLayer[],
   ElementwiseLayer[Ramp],
   PoolingLayer[{2, 2}, {2, 2}],
   ConvolutionLayer[32, {5, 5}],
   BatchNormalizationLayer[],
   ElementwiseLayer[Ramp],
   PoolingLayer[{2, 2}, {2, 2}],
   ConvolutionLayer[64, {5, 5}],
   BatchNormalizationLayer[],
   ElementwiseLayer[Ramp],
   PoolingLayer[{2, 2}, {2, 2}],
   ConvolutionLayer[48, {3, 3}],
   BatchNormalizationLayer[],
   ElementwiseLayer[Ramp],
   PoolingLayer[{2, 2}, {2, 2}],
   FlattenLayer[],
   DropoutLayer[],
   DotPlusLayer[500],
   ElementwiseLayer[Ramp],
   DotPlusLayer[2],
   SoftmaxLayer[]
  },
  "Output" → NetDecoder[{"Class", {"clean", "contaminated"}}],
  "Input" → NetEncoder[{"Image", {256, 256}(*,"Grayscale"*)}]
 ]
```

NetChain[

		image
	Input	3–tensor (size: 3 × 256 × 256)
1	ConvolutionLayer	3–tensor (size: 16 × 252 × 252)
2	BatchNormalizationLayer	3–tensor (size: 16 × 252 × 252)
3	Ramp	3–tensor (size: 16 × 252 × 252)
4	PoolingLayer	3–tensor (size: 16 × 126 × 126)
5	ConvolutionLayer	3–tensor (size: 32 × 122 × 122)
6	BatchNormalizationLayer	3–tensor (size: 32 × 122 × 122)
7	Ramp	3–tensor (size: 32 × 122 × 122)
8	PoolingLayer	3–tensor (size: 32 × 61 × 61)
9	ConvolutionLayer	3–tensor (size: 64 × 57 × 57)
10	BatchNormalizationLayer	3–tensor (size: 64 × 57 × 57)
11	Ramp	3–tensor (size: 64 × 57 × 57)
12	PoolingLayer	3–tensor (size: 64 × 28 × 28)
13	ConvolutionLayer	3–tensor (size: 48 × 26 × 26)
14	BatchNormalizationLayer	3–tensor (size: 48 × 26 × 26)
15	Ramp	3–tensor (size: 48 × 26 × 26)
16	PoolingLayer	3–tensor (size: 48 × 13 × 13)
17	FlattenLayer	vector (size: 8112)
18	DropoutLayer	vector (size: 8112)
19	LinearLayer	vector (size: 500)
20	Ramp	vector (size: 500)
21	LinearLayer	vector (size: 2)
22	SoftmaxLayer	vector (size: 2)
	Output	class

(uninitialized)

]

Train the neural net

```
net = NetTrain[net, dat[[tix]], ValidationSet → dat[[vix]], TargetDevice → "GPU",
   "Method" → {"ADAM", "L2Regularization" → 5, "InitialLearningRate" → 0.0001}];
```

Check out how we did

```
cm = ClassifierMeasurements[net, dat[[ttix]]];
```

```
cm["Accuracy"]
```

```
0.904823
```

```
cm["ConfusionMatrixPlot"]
```

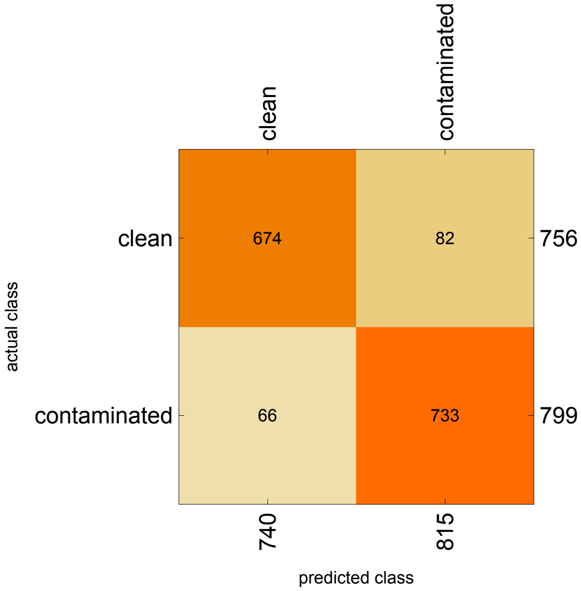

Check on some of the images that the neural net misclassified, in this case, clean samples that were misclassified as contaminated

```
Labeled[RandomChoice[cm["Examples" → {"clean", "contaminated"}], 10],
 "Clean Samples Predicted as Contaminated"]
```

{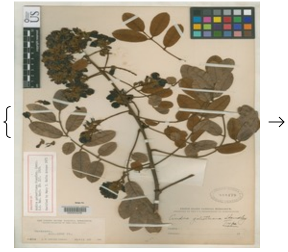 → clean, 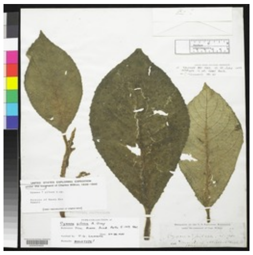 → clean,

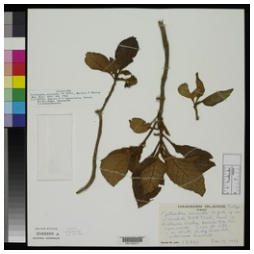 → clean, 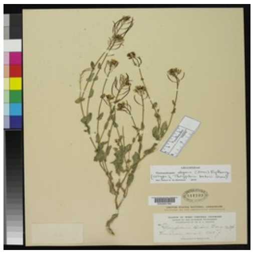 → clean,

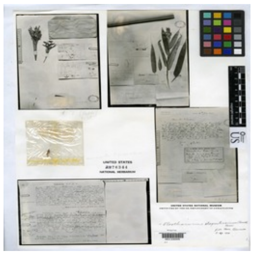 → clean, 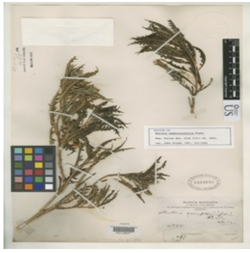 → clean, 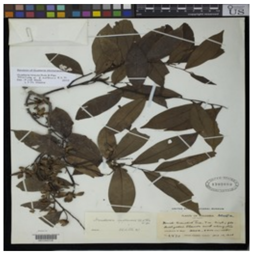 → clean,

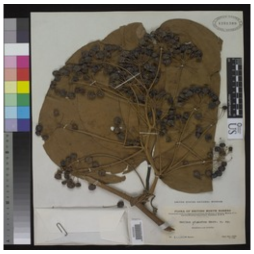 → clean, 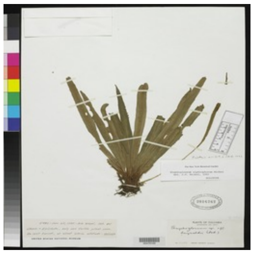 → clean, 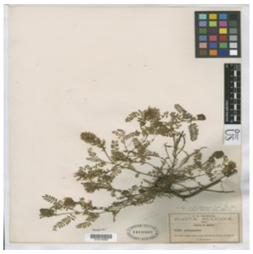 → clean}

Clean Samples Predicted as Contaminated

Now check out the contaminated samples that were classified as clean. Many have only very small evidence of contamination.

```
Labeled[RandomChoice[cm["Examples" → {"contaminated", "clean"}], 10],
 "Contaminated Samples Predicted as Clean"]
```

{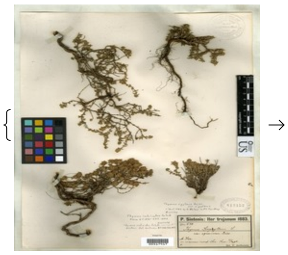 → contaminated, 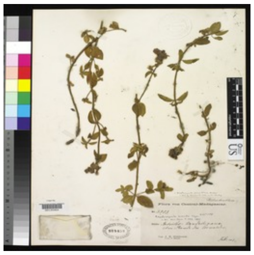 → contaminated,

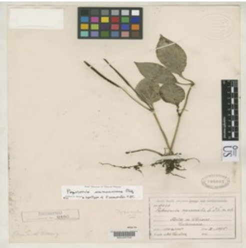 → contaminated, 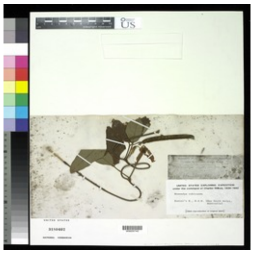 → contaminated,

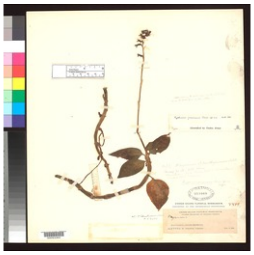 → contaminated, 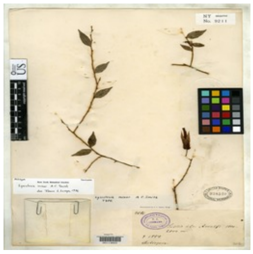 → contaminated,

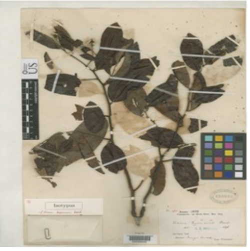 → contaminated, 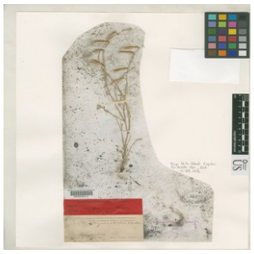 → contaminated,

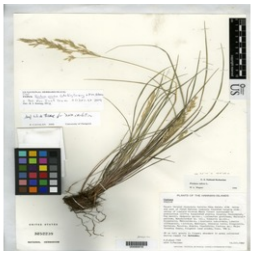 → contaminated, 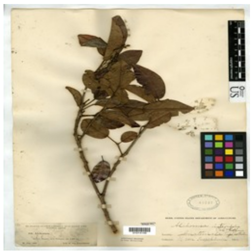 → contaminated}

Contaminated Samples Predicted as Clean

```
testDatClean = Select[dat[[ttix]], Values@# == "clean" &];
testDatContam = Select[dat[[ttix]], Values@# == "contaminated" &];
{Length@testDatClean, Length@testDatContam}
```

```
{756, 799}
```

```
pTestDatClean = net[Keys@testDatClean, {"Probability", "clean"}];
pTestDatContam = net[Keys@testDatContam, {"Probability", "clean"}];
```

```
Histogram[{pTestDatClean, pTestDatContam}, 20, GridLines → Automatic,
 GridLinesStyle → Directive[Dotted, Gray], Frame → True,
 FrameLabel → {"Probability of Clean"}, ChartLegends → {"Clean", "Contaminated"}]
```

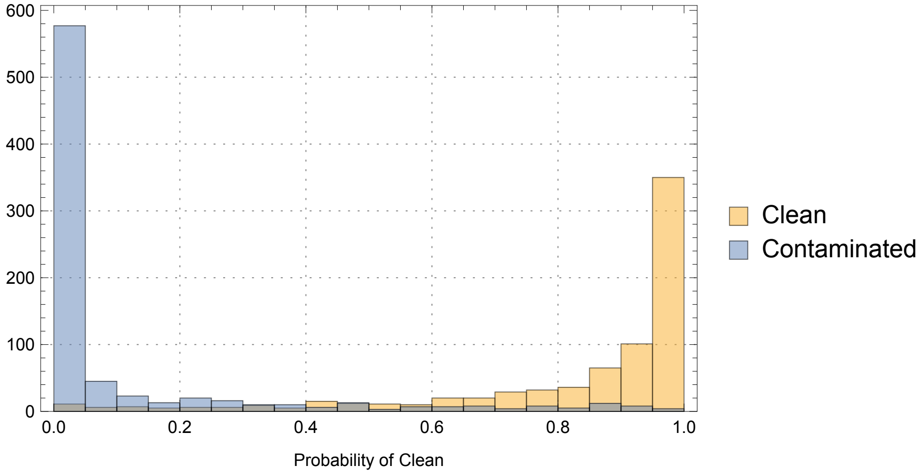


```
pL = SmoothKernelDistribution[pTestDatClean];
pO = SmoothKernelDistribution[pTestDatContam];
```

```
Plot[
 {PDF[pO, x], PDF[pL, x]}, {x, 0, 1},
 GridLines → Automatic,
 GridLinesStyle → Directive[Dotted, Gray],
 Frame → True,
 FrameLabel → {"Kernel Distribution, Prob(x,Clean)"},
 PlotLegends → {"Contaminated", "Clean"}
]
```

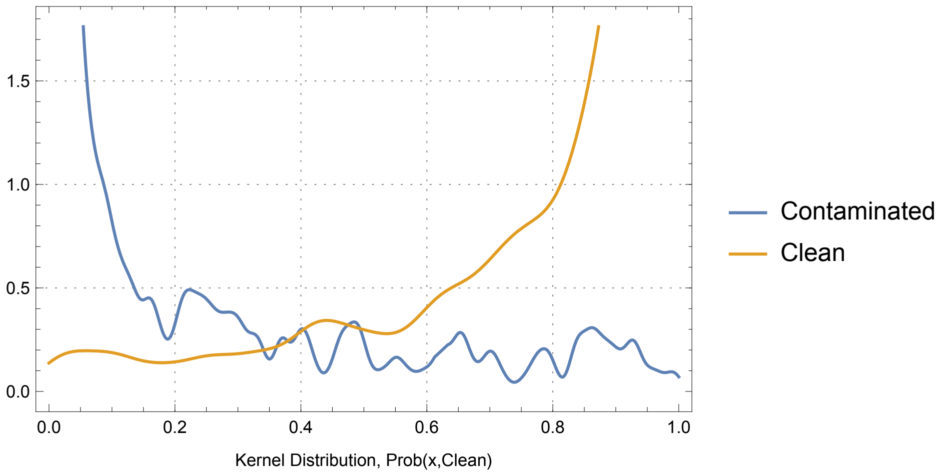


```
FindRoot[PDF[pL, x] == PDF[pO, x], {x, 0.58}]
{x → 0.474693}
```

```
Plot[
 {1 - CDF[pO, x], CDF[pL, x]},
 {x, 0, 1},
 GridLines → Automatic,
 GridLinesStyle → Directive[Dotted, Gray],
 Frame → True,
 FrameLabel → {"Kernel Distribution, Prob(x,clean)"},
 PlotLegends → {"Contaminated", "Clean"}
]
```

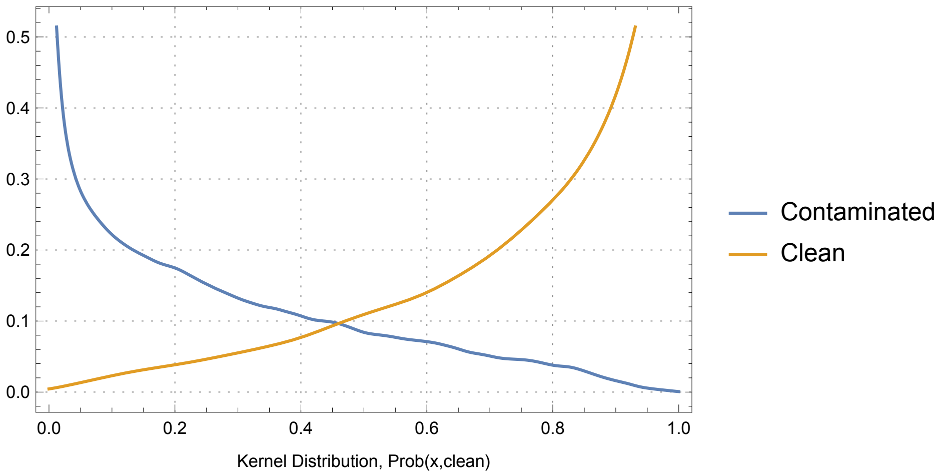


```
FindRoot[1 - CDF[pL, x] == CDF[pO, x], {x, 0.48}]
```

```
{x → 0.459614}
```

```
ptol = 0.459614;
{1 - CDF[pO, ptol], CDF[pL, ptol]}
```

```
{0.0964787, 0.0964788}
```

```
Total@%
```

```
0.192957
```

```
xx = Range[0.3, 0.9, 0.01];
f[x_] := Total[{1 - CDF[p0, x], CDF[pL, x]}];
xypoints = Transpose[{xx, f[xx]}];
ListPlot[xypoints]
```

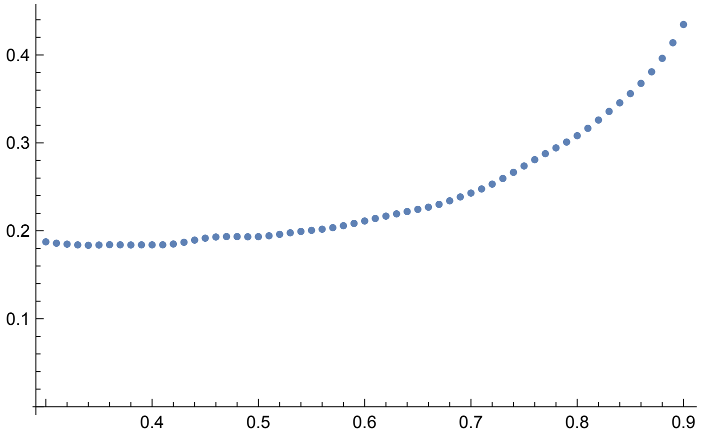


```
SortBy[xypoints, #[[2]] &] // First
{0.34, 0.183585}

ptol = 1 - 0.620703

0.379297`
0.379297

ptol = 0.459614
0.459614

cm["ConfusionMatrixPlot", IndeterminateThreshold → ptol]
```

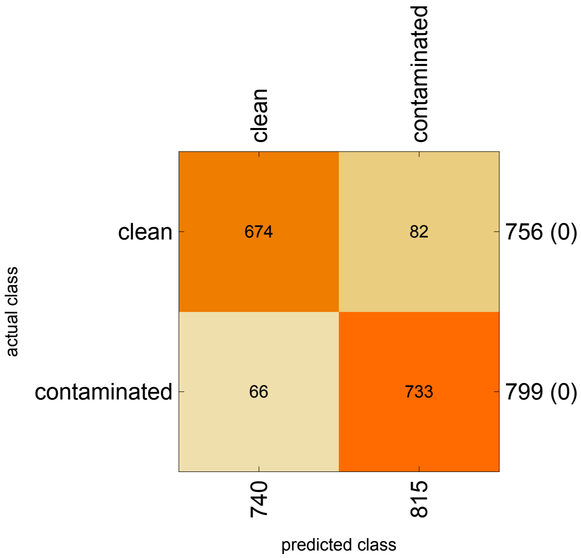

```
cm["Accuracy", IndeterminateThreshold → ptol]
```

0.904823

```
cm["FScore", IndeterminateThreshold → ptol]
```

⟨|clean → 0.90107, contaminated → 0.908302|⟩

```
cm["Error", IndeterminateThreshold → ptol]
```

0.0951768

```
cm["RejectionRate", IndeterminateThreshold → ptol]
```

0.

Generate rejection plot

```
Show[
 cm["AccuracyRejectionPlot"],
 GridLines -> Automatic,
 GridLinesStyle → Directive[Dotted, Gray]
]
```

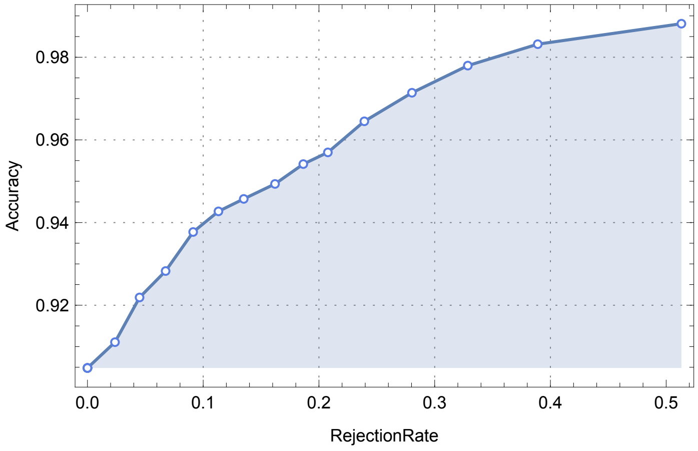

Save the trained neural net

```
networkdir = FileNameJoin[{root, "networks"}];
```

```
Export[FileNameJoin[{networkdir, "256_90.4_image.wlnet"}], net]
```

/pool/isilon/genomics/frandsenp/NVIDIA/Botany/Mathematica/networks/256_90.4_image.
wlnet
