# Supplementary material for: Applications of deep convolutional neural networks to digitized natural history collections
Source: Biodivers Data J. 2017 Nov 2;(5):e21139. doi: 10.3897/BDJ.5.e21139 (PMC5680669; doi:10.3897/BDJ.5.e21139)
Supplement: Supplementary material 4 — Annotated notebook used to define and train the clubmoss/spikemoss CNN [file bdj-05-e21139-s004.pdf]

Load the paths and the data

```
root = "/pool/isilon/dl_images";
datadirin = FileNameJoin[{root, "data"}];
networkdir = FileNameJoin[{root, "networks"}];
datadirLyco = FileNameJoin[{datadirin, "Lycopodiaceae/resized_256"}];
datadirSella = FileNameJoin[{datadirin, "Sellaginella/resized_256"}];
filesLyco = FileNames["*.jpg", datadirLyco];
filesSella = FileNames["*.jpg", datadirSella];
{Length@filesLyco, Length@filesSella}
classes = {"Lyco", "Sella"};
imgsz = {256, 256};
```

```
{9276, 9131}
```

Load the GPU components, fix Java for the Linux machine

```
Needs["CUDALink`"]
```

```
<< JLink`;
InstallJava[];
ReinstallJava[JVMArguments → "-Xmx10g"];
```

Import the data

```
datLyco = ParallelMap[Import[#] &, filesLyco];
datSella = ParallelMap[Import[#] &, filesSella];
```

Take a look at a few images to ensure they look right

```
RandomChoice@datLyco
```

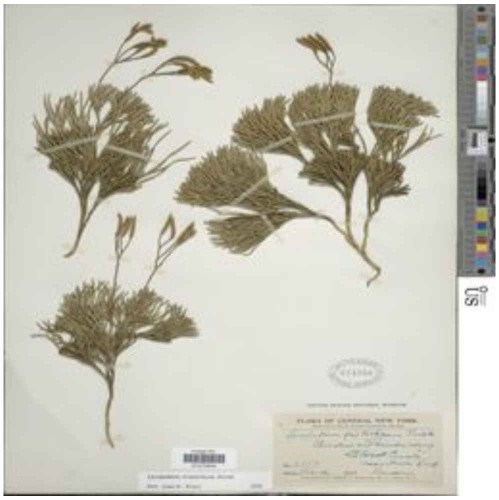

```
RandomChoice@datSella
```

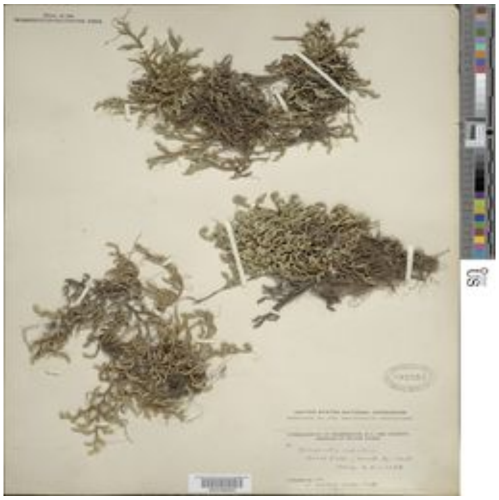

Make sure that all of the images loaded properly

```
{Dimensions@datLyco, Dimensions@datSella}
```

```
{{9276}, {9131}}
```

Flatten and annotate the image samples

```
dat = RandomSample@Join[Thread[datLyco → "Lyco"], Thread[datSella → "Sella"]];
Dimensions@dat
```

```
{18407}
```

Take another look to make sure that they're properly labelled

```
RandomSample[dat, 30]
```

{ 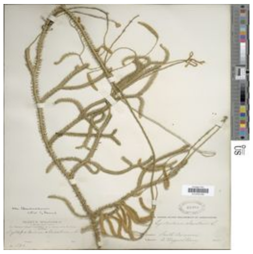 → Lyco, 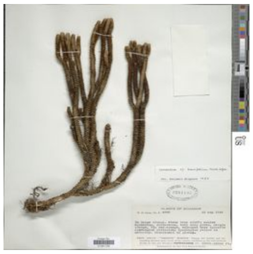 → Lyco, 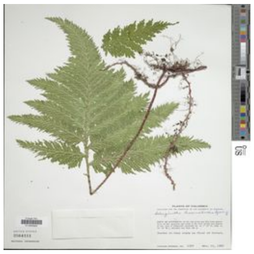 → Sella,

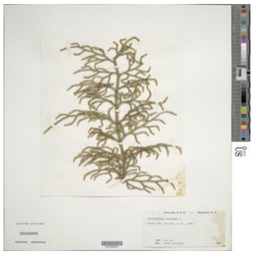 → Lyco, 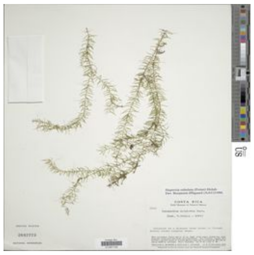 → Lyco, 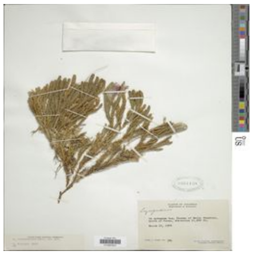 → Lyco,

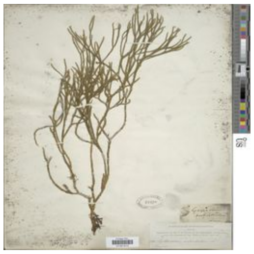 → Lyco, 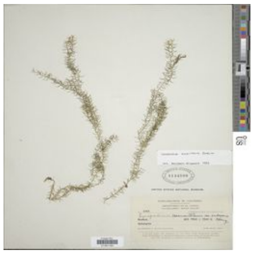 → Lyco, 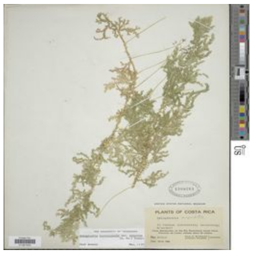 → Sella,

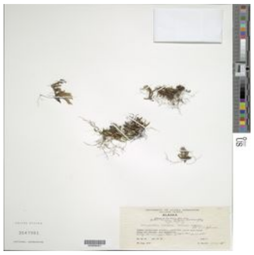 → Sella, 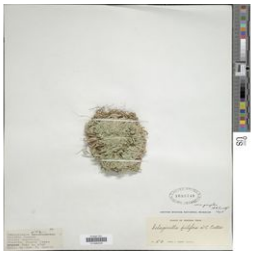 → Sella, 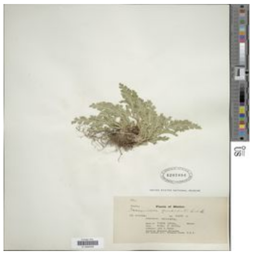 → Sella,

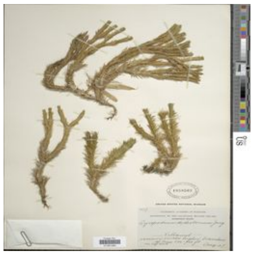 → Lyco, 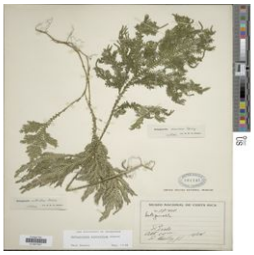 → Sella, 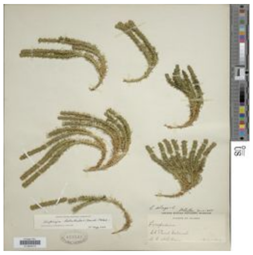 → Lyco,

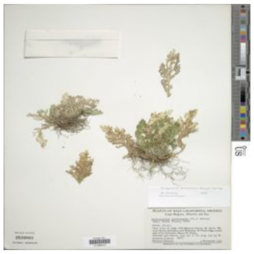 → Sella, 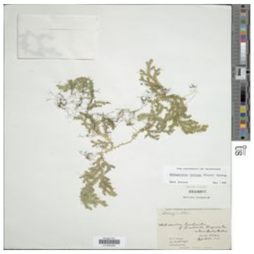 → Sella, 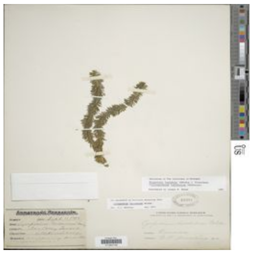 → Lyco,

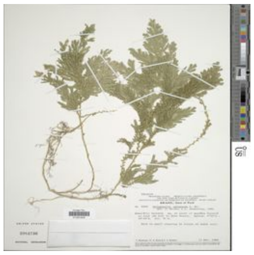 → Sella, 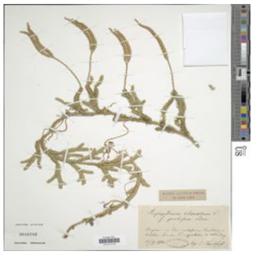 → Lyco, 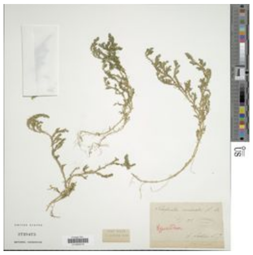 → Sella,

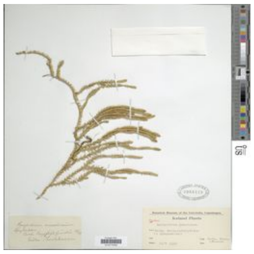 → Lyco, 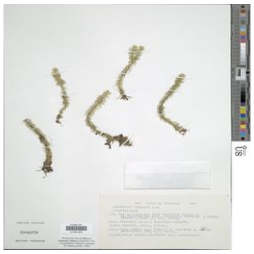 → Lyco, 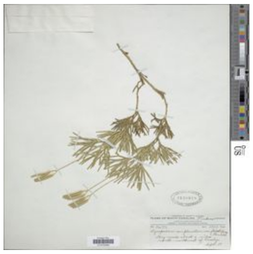 → Lyco,

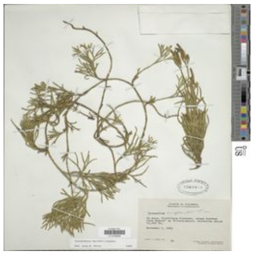 → Lyco, 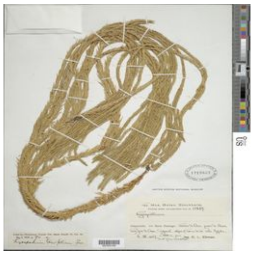 → Lyco, 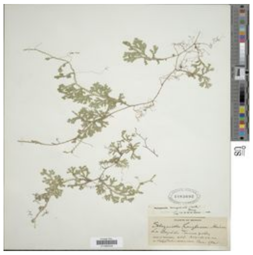 → Sella,

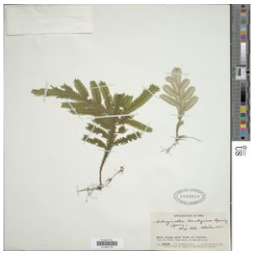 → Sella, 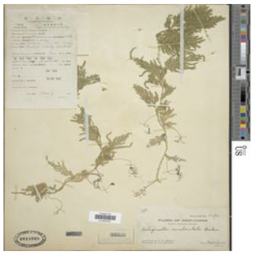 → Sella, 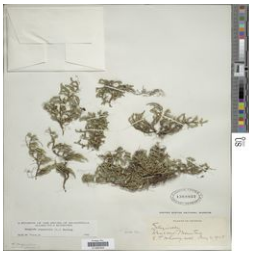 → Sella}

Estimate entropies

```
lycoEntropy = ParallelMap[ImageMeasurements[#, "Entropy"] &, datLyco];
sellaEntropy = ParallelMap[ImageMeasurements[#, "Entropy"] &, datSella];
```

See what the entropy distributions look like

```
Histogram[{lycoEntropy, sellaEntropy}, 40, ChartLegends → {"Lyco", "Sella"},
 GridLines → Automatic, GridLinesStyle → Directive[Dotted, Gray],
 Frame → True, FrameLabel → {Style["Entropy", FontSize → 14]}]
```

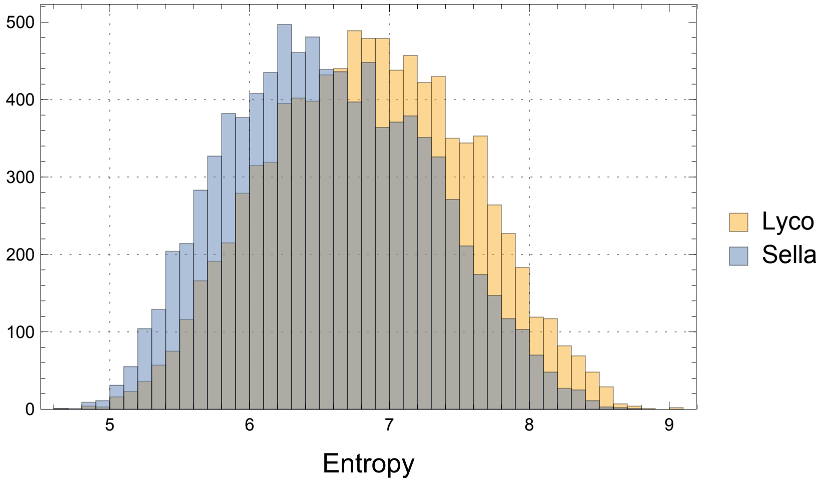


```
Plot[{CDF[EmpiricalDistribution[Map[Mean, Partition[lycoEntropy, 108]]], x],
  CDF[EmpiricalDistribution[Map[Mean, Partition[sellaEntropy, 108]]], x]},
 {x, 6.0, 7.5}, PlotLegends → {"Lyco", "Sella"}, GridLines → Automatic,
 GridLinesStyle → Directive[Dotted, Gray], Frame → True,
 FrameLabel → {Style["Entropy", FontSize → 14]}]
```

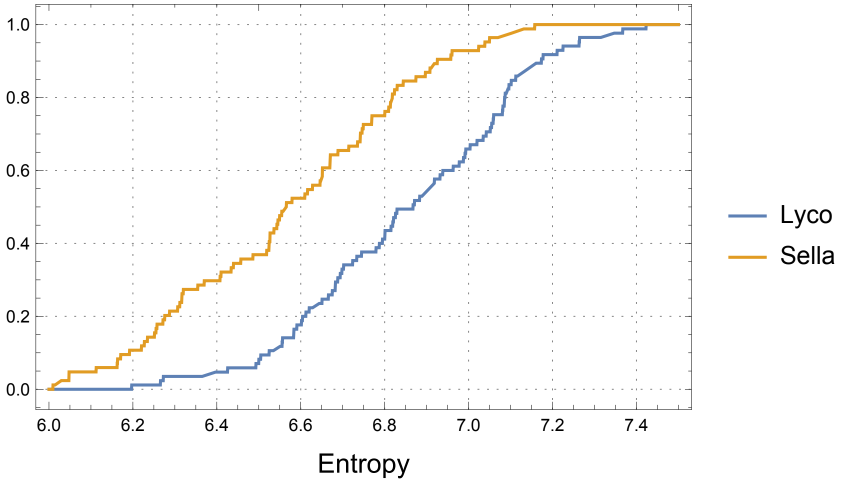

Randomly order images, then generate training data set (70% of data, “tix”), validation data set (20% of data, “vix”), and test data set (10% of data, “ttix”). Note that the test data set will not be used for training on the neural net.

```
rs = RandomSample[Range[Length[dat]]];
tix = rs[[1 ;; Round[Length[dat] * 0.7]]];
vix = rs[[Round[Length[dat] * 0.7] + 1 ;; Round[Length[dat] * 0.9]]];
ttix = rs[[Round[Length[dat] * 0.9] + 1 ;;]];
{Length@tix, Length@vix, Length@ttix}
```

{12 885, 3681, 1841}

Generate architecture for untrained net

```
net = NetChain[
  {
   ConvolutionLayer[10, {5, 5}],
   BatchNormalizationLayer[],
   ElementwiseLayer[Ramp],
   PoolingLayer[{2, 2}, "Stride" → 2],
   ConvolutionLayer[40, {5, 5}],
   BatchNormalizationLayer[],
   ElementwiseLayer[Ramp],
   PoolingLayer[{2, 2}, "Stride" → 2],
   FlattenLayer[],
   DropoutLayer[],
   DotPlusLayer[500],
   ElementwiseLayer[Ramp],
   DotPlusLayer[2],
   SoftmaxLayer[]
  },
  "Input" → NetEncoder[{"Image", imgsz(*,"Grayscale"*)}],
  "Output" → NetDecoder[{"Class", classes}]
 ]
```

NetChain[

		image
	Input	3–tensor (size: 3 × 256 × 256)
1	ConvolutionLayer	3–tensor (size: 10 × 252 × 252)
2	BatchNormalizationLayer	3–tensor (size: 10 × 252 × 252)
3	Ramp	3–tensor (size: 10 × 252 × 252)
4	PoolingLayer	3–tensor (size: 10 × 126 × 126)
5	ConvolutionLayer	3–tensor (size: 40 × 122 × 122)
6	BatchNormalizationLayer	3–tensor (size: 40 × 122 × 122)
7	Ramp	3–tensor (size: 40 × 122 × 122)
8	PoolingLayer	3–tensor (size: 40 × 61 × 61)
9	FlattenLayer	vector (size: 148840)
10	DropoutLayer	vector (size: 148840)
11	LinearLayer	vector (size: 500)
12	Ramp	vector (size: 500)
13	LinearLayer	vector (size: 2)
14	SoftmaxLayer	vector (size: 2)
	Output	class

(uninitialized)

]

Train the neural net

```
net = NetTrain[net, dat[[tix]], ValidationSet → dat[[vix]], TargetDevice → "GPU",
   Method → {"ADAM", "L2Regularization" → 5, "InitialLearningRate" → 0.0001}];
```

See how well it did

```
cm = ClassifierMeasurements[net, dat[[ttix]]];
```

Check accuracy based on test data that was not used in training

```
cm["Accuracy"]
```

```
0.955459
```

```
cm["ConfusionMatrixPlot"]
```

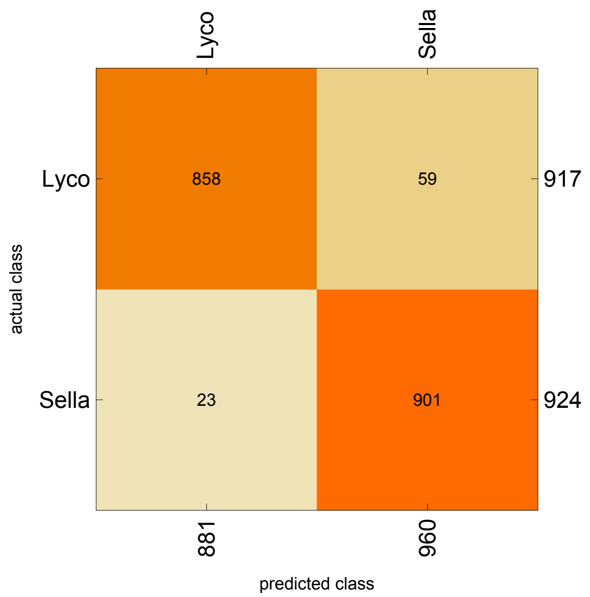


```
testDatLyco = Select[dat[[ttix]], Values@# == "Lyco" &];
testDatSella = Select[dat[[ttix]], Values@# == "Sella" &];
{Length@testDatLyco, Length@testDatSella}
```

```
{917, 924}
```

```
pTestDatLyco = net[Keys@testDatLyco, {"Probability", "Lyco"}];
pTestDatSella = net[Keys@testDatSella, {"Probability", "Lyco"}];
```

Visualize the probabilities

```
Histogram[{pTestDatLyco, pTestDatSella}, 20, GridLines → Automatic,
 GridLinesStyle → Directive[Dotted, Gray], Frame → True,
 FrameLabel → {"Probability of Lyco"}, ChartLegends → {"Lyco", "Sella"}]
```

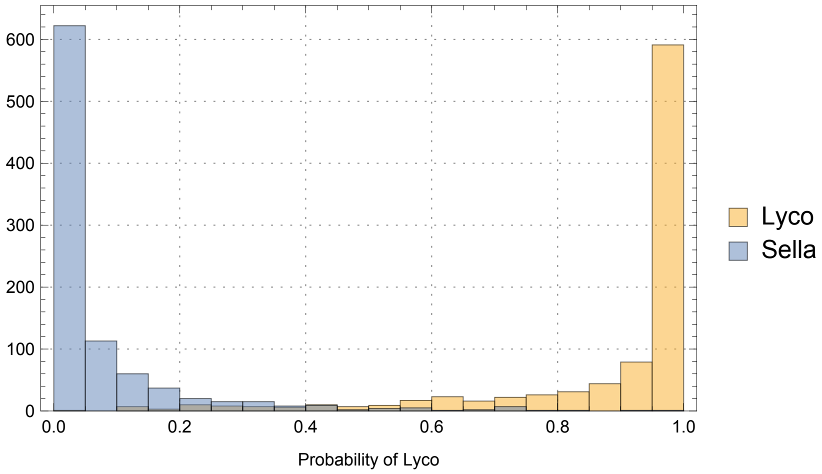


```
pL = SmoothKernelDistribution[pTestDatLyco];
pS = SmoothKernelDistribution[pTestDatSella];
```

```
Plot[{PDF[pS, x], PDF[pL, x]}, {x, 0, 1}, GridLines → Automatic,
 GridLinesStyle → Directive[Dotted, Gray], Frame → True,
 FrameLabel → {"Kernel Distribution, Prob(x,Lyco)"},
 PlotLegends → {"Sella", "Lyco"}]
```

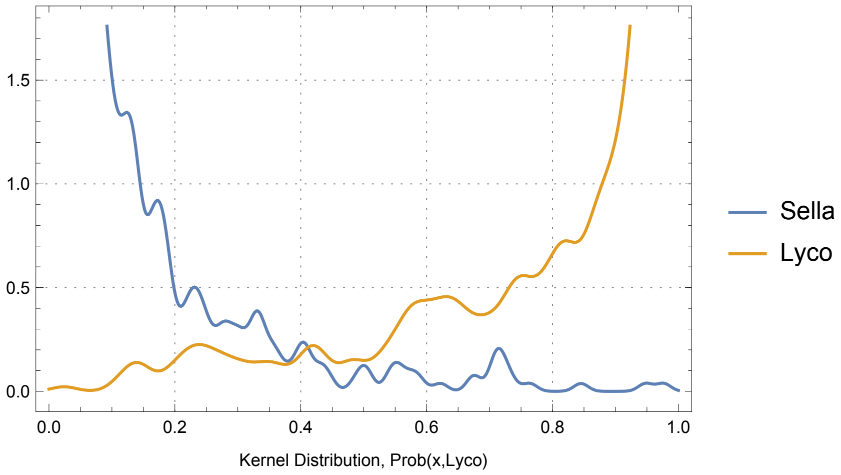


```
FindRoot[PDF[pL, x] == PDF[pS, x], {x, 0.4}]
{x → 0.411552}
```

```
Plot[{(*Used 1 minus the CDF so that the plots overlap*)
   1 - CDF[pS, x], CDF[pL, x]}, {x, 0, 1}, GridLines → Automatic,
  GridLinesStyle → Directive[Dotted, Gray], Frame → True,
  FrameLabel → {"Kernel Distribution, Prob(x,Lyco)"},
  PlotLegends → {"Sella", "Lyco"}]
```

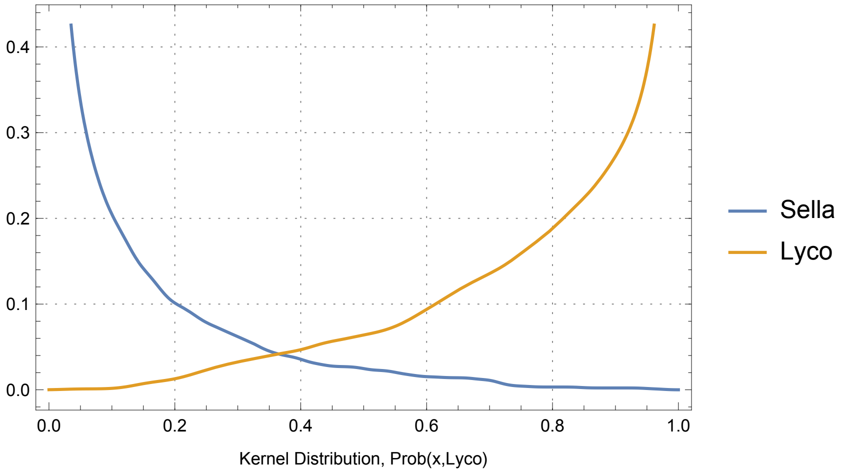


```
ptol = 0.411552;
{1 - CDF[pS, ptol], CDF[pL, ptol]}
```

{0.0329841, 0.0490816}

```
Total@%
```

0.0820657

```
xx = Range[0.3, 0.8, 0.01];
f[x_] := Total[{1 - CDF[pS, x], CDF[pL, x]}];
xypoints = Transpose[{xx, f[xx]}];
ListPlot[xypoints]
```

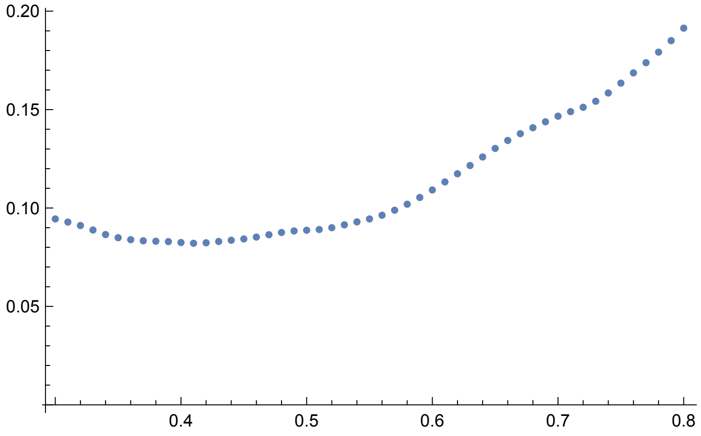


```
SortBy[xypoints, #[[2]] &] // First
```

{0.41, 0.0820751}

```
cm["ConfusionMatrixPlot", IndeterminateThreshold → ptol]
```

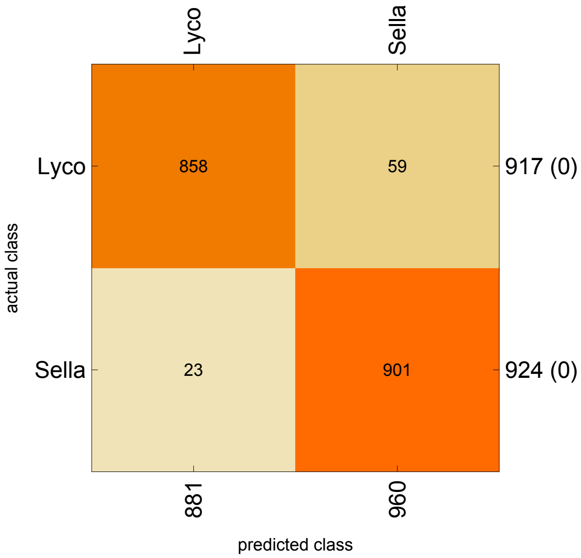


```
ptol = 1 - 0.411552
0.588448
```

```
cm["ConfusionMatrixPlot", IndeterminateThreshold → ptol]
```

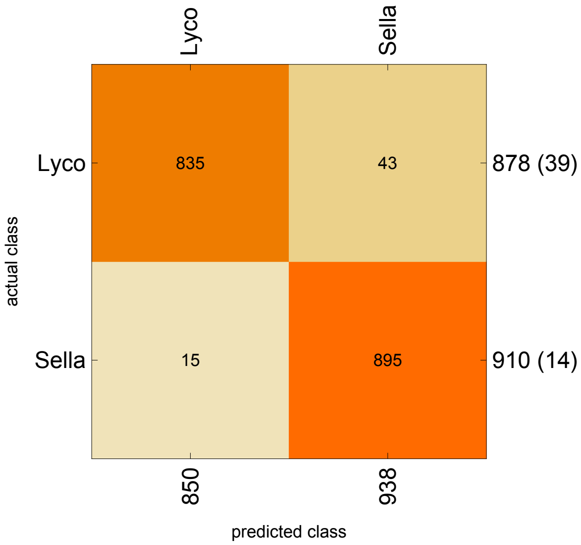


```
cm["Accuracy", IndeterminateThreshold → ptol]
0.967562
```

```
cm["FScore", IndeterminateThreshold → ptol]
⟨|Lyco → 0.966435, Sella → 0.968615|⟩
```

```
cm["Error", IndeterminateThreshold → ptol]
0.0324385
```

```
cm["RejectionRate", IndeterminateThreshold → ptol]
```

```
0.0287887
```

Generate rejection plot

```
Show[cm["AccuracyRejectionPlot"],
 GridLines → Automatic, GridLinesStyle → Directive[Dotted, Gray]]
```

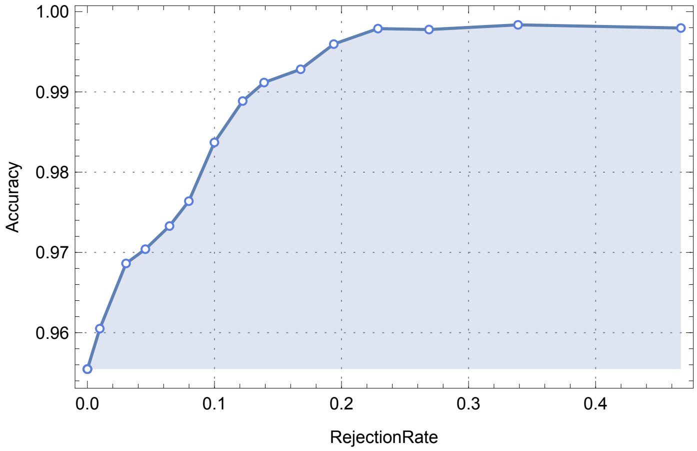

Save network

```
Export[FileNameJoin[{networkdir, "lycosella_256_clr.wlnet"}], net]
```

```
/scratch/genomics/frandsenp/NVIDIA/LycoSella/Mathematica/networks/lycosella_256
  _clr.wlnet
```

```
networkdir = "/scratch/genomics/frandsenp/NVIDIA/LycoSella/Mathematica/networks"
```

```
/scratch/genomics/frandsenp/NVIDIA/LycoSella/Mathematica/networks
```
